# Phenotyping Hepatic Immune-Related Adverse Events in the Setting of Immune Checkpoint Inhibitor Therapy

**DOI:** 10.1200/CCI.23.00159

**Published:** 2024-05-10

**Authors:** Theodore C. Feldman, David E. Kaplan, Albert Lin, Jennifer La, Jerry S.H. Lee, Mayada Aljehani, David P. Tuck, Mary T. Brophy, Nathanael R. Fillmore, Nhan V. Do

**Affiliations:** ^1^ VA Boston Healthcare System, Boston, MA; ^2^Harvard Medical School, Boston, MA; ^3^ Corporal Michael J. Crescenz Department of Veterans Affairs Medical Center, Philadelphia, PA; ^4^ Perelman School of Medicine at the University of Pennsylvania Medical School, Philadelphia, PA; ^5^ VA Palo Alto Healthcare System, Palo Alto, CA; ^6^ Stanford University School of Medicine, Stanford, CA; ^7^ Ellison Institute of Technology, Los Angeles, CA; ^8^ Department of Medicine, Keck School of Medicine, University of Southern California, Los Angeles, CA; ^9^ Department of Chemical Engineering and Materials Sciences, Viterbi School of Engineering, University of Southern California, Los Angeles, CA; ^10^ Department of Quantitative and Computational Biology, Dornsife College of Letters, Arts and Sciences, University of Southern California, Los Angeles, CA; ^11^ Boston University Chobanian & Avedisian School of Medicine, Boston, MA; ^12^ Dana-Farber Cancer Institute, Boston, MA

## Abstract

**PURPOSE:**

We present and validate a rule-based algorithm for the detection of moderate to severe liver-related immune-related adverse events (irAEs) in a real-world patient cohort. The algorithm can be applied to studies of irAEs in large data sets.

**METHODS:**

We developed a set of criteria to define hepatic irAEs. The criteria include: the temporality of elevated laboratory measurements in the first 2-14 weeks of immune checkpoint inhibitor (ICI) treatment, steroid intervention within 2 weeks of the onset of elevated laboratory measurements, and intervention with a duration of at least 2 weeks. These criteria are based on the kinetics of patients who experienced moderate to severe hepatotoxicity (Common Terminology Criteria for Adverse Events grades 2-4). We applied these criteria to a retrospective cohort of 682 patients diagnosed with hepatocellular carcinoma and treated with ICI. All patients were required to have baseline laboratory measurements before and after the initiation of ICI.

**RESULTS:**

A set of 63 equally sampled patients were reviewed by two blinded, clinical adjudicators. Disagreements were reviewed and consensus was taken to be the ground truth. Of these, 25 patients with irAEs were identified, 16 were determined to be hepatic irAEs, 36 patients were nonadverse events, and two patients were of indeterminant status. Reviewers agreed in 44 of 63 patients, including 19 patients with irAEs (0.70 concordance, Fleiss' kappa: 0.43). By comparison, the algorithm achieved a sensitivity and specificity of identifying hepatic irAEs of 0.63 and 0.81, respectively, with a test efficiency (percent correctly classified) of 0.78 and outcome-weighted F1 score of 0.74.

**CONCLUSION:**

The algorithm achieves greater concordance with the ground truth than either individual clinical adjudicator for the detection of irAEs.

## Teaser

Three (sometimes 4) rules to identify hepatic immune-related adverse events in the setting of ICI.

## Graphical abstract

**Figure:**
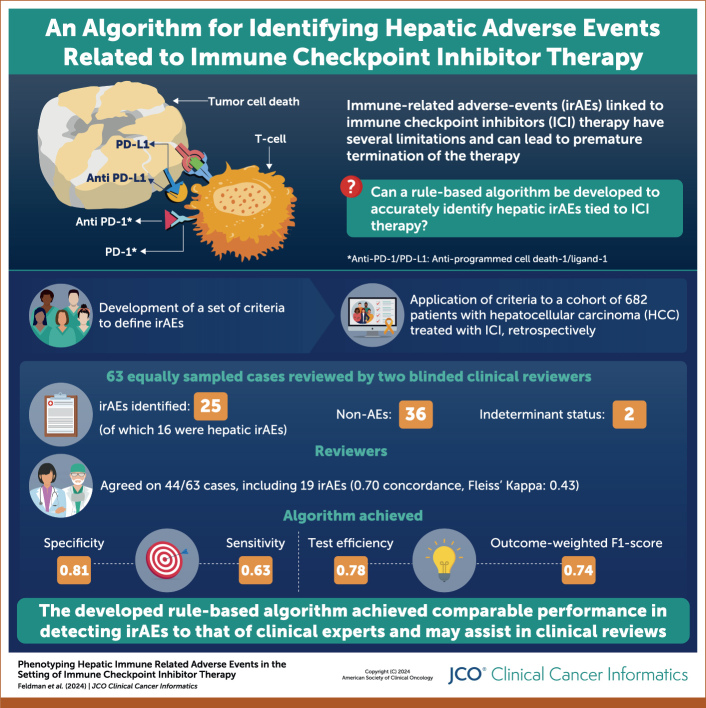

## INTRODUCTION

Immune-related adverse events (irAEs) associated with immune checkpoint inhibitor (ICI) therapy can lead to premature termination of therapy, reduce quality of life, cause organ damage, and can be fatal. irAEs typically last longer and present later than most adverse events associated with chemotherapy or targeted inhibitors.^1^ ICI-mediated hepatitis is one of the most frequent adverse events and occurs at incidences ranging from 0.3% to 37%.^2-7^

**CONTEXT**

**Key Objective**
To develop a rule-based computable phenotype to identify hepatic adverse events in the setting of immune checkpoint inhibitor (ICI) therapy based on timing of onset, timing of treatment, and duration of treatment in patients treated for hepatocellular carcinoma.
**Knowledge Generated**
We developed a set of criteria to define hepatic immune-related adverse events (irAEs). The criteria include: the temporality of elevated laboratory measurements in the first 2-14 weeks of ICI treatment, steroid intervention within 2 weeks of the onset of elevated laboratory measurements, and intervention with a duration of at least 2 weeks. These criteria are based on the kinetics of patients who experienced moderate to severe hepatotoxicity (Common Terminology Criteria for Adverse Events grades 2-4). We demonstrate performance comparable with the agreement between two expert reviewers.
**Relevance *(J.L. Warner)***
An algorithm capable of retrospectively identifying hepatic irAEs in the setting of ICIs based on routinely obtained data is valuable for research as well as the phase IV setting.*
  *Relevance section written by *JCO Clinical Cancer Informatics* Editor-in-Chief Jeremy L. Warner, MD, MS, FAMIA, FASCO.

Improvements to irAE management are needed, including the need for better approaches to identify irAEs, predictive biomarkers, and real-world measures of the incidences and severity of irAEs to alleviate burden placed on care teams supporting ICI-treated patients.^1,2,5,6,8-11^ Infrastructure and approaches to report irAEs in real-world settings remain under development and are less standardized than in clinical trials.^12,13^

Direct drug-induced liver toxicity is identified using validated instruments, such as the Roussel-UCLAF Causality Assessment Method (RUCAM).^14^ The RUCAM provides a highly sensitive, highly specific detection instrument when used by clinical domain experts to evaluate individual patients. However, the RUCAM is intended to be applied at the level of the individual patients and can suffer from reduced performance because of subjectivity and reliability.^15^ An electronic update to the RUCAM, the Revised Electronic Causality Assessment Method (RECAM) was recently reported based on registry data, providing similar performance to the RUCAM.^15^ Both the RUCAM and RECAM focus on clinical diagnosis. The presentation of liver injury is heterogenous and may vary greatly with the type of medication and patient population. In particular, irAEs associated with ICI therapies may present over a prolonged duration following initiation.^15^ As such, ICI-specific tools to identify hepatic irAEs are needed.

Here, we present and validate a rule-based algorithm computable phenotype for the detection of moderate to severe liver-related irAEs using longitudinal patient-level electronic health record (EHR) data for hepatic irAEs in the setting of ICI therapy. Our phenotype is intended to identify hepatic irAEs in retrospective, real-world, large-scale clinical data where individual chart review is not feasible in a cohort of patients with hepatocellular carcinoma (HCC) and underlying chronic liver disease where biopsies are unlikely to be performed. We desire to use minimal features for ease of use and to facilitate future predictive modeling where the use of high-dimensional variable space may introduce sparseness and create selection bias. Our phenotype is built upon considerations of the RUCAM/RECAM criteria. We use longitudinal patient-level EHR data, particularly changes in laboratory values and the chronology of ICI treatment and medications associated with the treatment of moderate to severe irAEs that are clinically actionable. Using this approach, we provide a framework to test other associations between irAEs, patient characteristics, and set of chronologic criteria by which to facilitate physician review of potential irAEs.

## METHODS

We conducted a retrospective study using data derived from a national cohort spanning patients in the Veterans Health Administration (VHA).^16^ We obtained electronic data on all patients who initiated ICI treatment in the US Department of Veterans Affairs (VA) system using the VA Corporate Data Warehouse, a national, continually updated repository of VHA electronic health records developed specifically to facilitate research.^17^ The study was approved by the Research and Development Committee and Institutional Review Board of the VA Boston Healthcare System and received a waiver of informed consent because the study presented minimal risk and could not practicably be conducted without a waiver.

**FIG 1.**
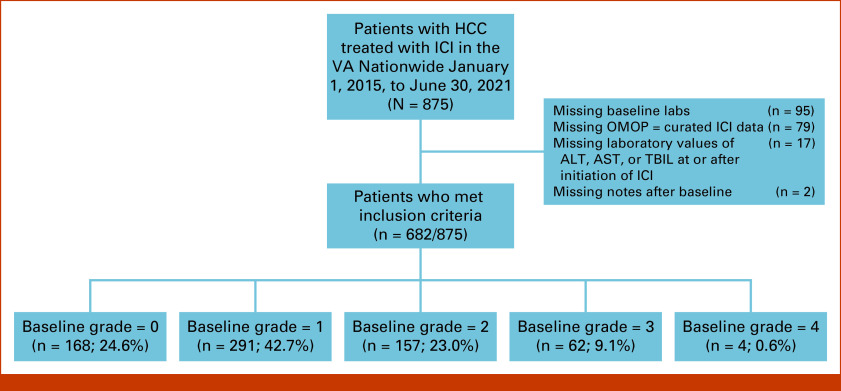
Flow diagram of the HCC cohort studied. Six hundred eighty-two patients treated with ICI for HCC met inclusion criteria. Patients are stratified by their maximum baseline grade of either ALT, AST, or TBIL. HCC, hepatocellular carcinoma; ICI, immune checkpoint inhibitor; OMOP, Observational Medical Outcomes Partnership; TBIL, bilirubin; VA, US Department of Veterans Affairs.

Our cohort comprised patients diagnosed with HCC and treated with ICI. Patients with HCC were identified using a previously validated algorithm between January 1, 2015, and June 30, 2021 (Fig 1).^18^ Relevant ICI therapies were adjudicated from a list of therapies derived from the HemOnc ontology.^19^ We defined the date of first ICI as the earliest ICI order date and took this to be the index date for this study.

We restricted our study to patients with at least one baseline laboratory measurement of ALT, AST, or bilirubin (TBIL) within 42 days before the initiation of ICI therapy (see the Data Supplement, Methods: Baseline laboratory value determination and Consideration of RUCAM/RECAM criteria and determination of time cutoffs, and the Data Supplement, Results: Data Supplement, Fig S1 and Table S1, for additional details). We all required all included patient to have relevant therapy orders presented in the VA instantiation of the Observational Medical Outcomes Partnership (OMOP) data model^20^ and to have at least one hematology-oncology progress note to facilitate chart review.

We considered the criteria of the RUCAM/RECAM in conjunction with expert clinical domain opinion as well as role of patient history and the influence of comorbidities, which may elevate ALT, AST, or TBIL directly through presentation or indirectly as the result of associated medications using a phecode-only PheWAS (see the Data Supplement, Methods: Analysis of comorbidities and patient-informed kinetics and Consideration of RUCAM/RECAM criteria and determination of time cutoffs, and the Data Supplement, Results: Data Supplement, Figs S2 and S3 and Table S2, for additional details).

**FIG 2.**
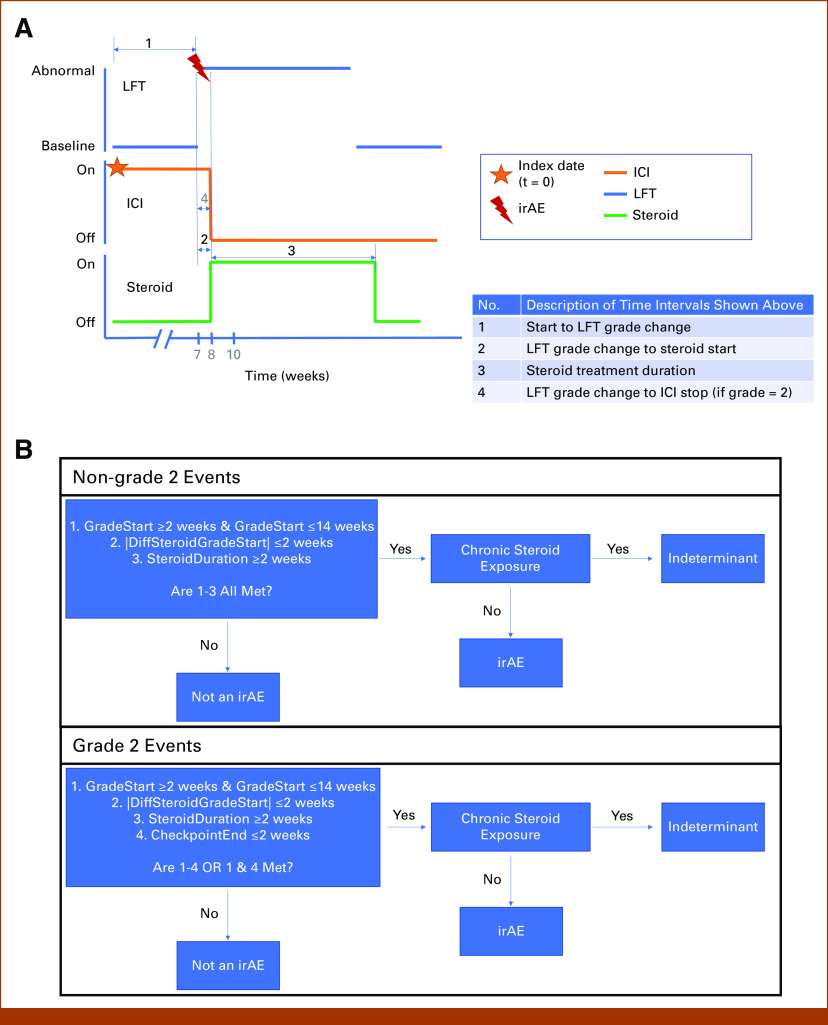
irAE detection algorithm. (A) A conceptual example of the time intervals considered in our hepatic irAE phenotype. These include: the time intervals between elevations in ALT, AST, and/or TBIL, ICI initiation/end, the introduction of steroid treatment, and the duration of steroid treatment. In this example, the irAE occurred at week 7 after ICI initiation. Here, steroid therapy was initiated for more than 2 weeks, and both steroid initiation and cessation of ICI therapy occurred within 2 weeks (in this example, within 1 week) after the elevation of the ALT/AST/TBIL lab. (B) Our phenotype definitions depending upon CTCAE grade of the potential event. (1) For all grades, an elevation in ALT, AST, or TBIL is determined to be an irAE if (a) it occurs between 2 and 14 weeks before the initiation of ICI or most recent cycle of ICI, (b) steroid treatment is given within 2 weeks of the elevation, and (c) for a duration of at least 2 weeks provided that the patient has no history of chronic steroid exposure. If these conditions are met, but the patient has a history of chronic steroid exposure, the elevation of ALT, AST, and/or TBIL is deemed to be of indeterminant status. If any of these three conditions are unmet, the elevation of ALT, AST, and/or TBIL is deemed to be a non-irAE. (2) For grade 2 events, we also deem an elevation in ALT, AST, or TBIL to be an irAE if (a) it occurs between 2 and 14 weeks before the initiation of ICI or most recent cycle of ICI and (b) ICI therapy is stopped within 2 weeks of the event. CTCAE, Common Terminology Criteria for Adverse Events; ICI, immune checkpoint inhibitor; irAE, immune-related adverse event; LFT, liver-associated blood test; TBIL, bilirubin.

We identified patients with baseline chronic steroid exposure defined as exposure before ICI with an exposure era lasting longer than 90 days (n = 8 patients) in the 6 months before the initiation of ICI (see the Data Supplement, Methods: Chronic steroid exposure, for additional details). For patients who experience changes in ALT, AST, and/or TBIL grades after the initiation of ICI, we calculate the time differences between the ICI cycle start/stop, steroid interval start/stop, and the change in ALT/AST/TBIL grade start/stop (Fig 2).

We selected the time cutoffs upon the reported time course of hepatic irAEs and clinical practice to identify irAEs^6^ and the observed kinetics of laboratory value elevations, and steroid treatments in our patient cohort (see the Data Supplement, Methods: Analysis of comorbidities and patient-informed kinetics, and Consideration of RUCAM/RECAM criteria and determination of time cutoffs, and the Data Supplement, Table S2, for additional details). We first grade elevations of ALT, AST, and/or TBIL. After assigning each liver enzyme elevation a Common Terminology Criteria for Adverse Events (CTCAE) grade, we iterate through each elevation present in the patient's EHR. We classify any elevation as an acute hepatic irAE if the following criteria are met (summarized in Fig 2B).

For events other than CTCAE grade 2:

1. It begins 2-14 weeks after the initiation of ICI therapy. Or, for elevations that occur after the first cycle of ICI, it begins 2-14 weeks after the initiation of the most recent cycle of ICI therapy.
2. Steroid treatment is initiated within 2 weeks of the start of the ALT, AST, and/or TBIL elevation.
3. Steroid duration is at least 2 weeks.

For events associated with CTCAE grade 2:

1. It begins 2-14 weeks after the initiation of ICI therapy. Or, for elevations that occur after the first cycle of ICI, it begins 2-14 weeks after the initiation of the most recent cycle of ICI therapy.
2. Steroid treatment is initiated within 2 weeks of the start of the ALT, AST, and/or TBIL elevation.
3. Steroid duration is at least 2 weeks.
4. Criteria 1 is met and ICI therapy is stopped within 2 weeks of the date of the event whether criteria 2 and 3 are met.

Regardless of grade, if all criteria are satisfied, but the patient has been identified as having baseline chronic exposure to steroids, the event is classified as indeterminant, otherwise the elevation is classified with non-irAE status.

We assessed the performance of our initial detection algorithm against the results of clinical expert chart review (see the Data Supplement, Methods: Chart review, for additional details) and took the consensus as the ground truth. Multiclass algorithm classification performance was then assessed by comparing the algorithm classification to the label determined by chart review on a per-class basis. The confusion matrix was determined for each class and then specificity, sensitivity, positive predictive value, negative predictive value, and accuracy were computed (see the Data Supplement, Methods: Confusion matrix definitions and Multiclass algorithm performance, for additional details).

We also determined patient characteristics stratified by baseline grade based on EHR data (see the Data Supplement, Methods: Baseline patient characteristics, for additional details).

To demonstrate potential applications of our algorithm for population-scale studies, we calculated the incidence of immune-mediated hepatitis by type and compared univariate relationships between baseline elevations of laboratory values, previous treatment with tyrosine kinase inhibitors (TKIs), median peak eosinophil to leukocyte ratio (ELR), median peak neutrophil to lymphocyte ratio (NLR) during 0-14 weeks of treatment, and the intrapatient variability of ELR and NLR between those patients deemed to have experienced an irAE versus those who deemed to not have experienced an irAE (see the Data Supplement, Methods: Calculations for downstream applications of the irAE phenotype to prior TKI exposure and derived ELR and NLR, for additional details).

For all categorical variables with counts, *P* values were calculated using Pearson's chi-squared test for count data as implemented in the *stats* R package, while continuous variables, including age and Charlson score, were calculated using the Kruskal-Wallis test.^21^

## RESULTS

We identified 682 patients overall diagnosed with HCC and treated with ICI between January 1, 2015, and June 30, 2021, who met our inclusion criteria (Fig 1). Patient characteristics are described in Table 1 (see the Data Supplement, Results: Extended patient characteristics, for additional details). We considered history of previous decompensation events associated with cirrhosis.^22,23^ A statistically significant greater proportion of patients with one or more baseline laboratory value equivalent to grade 1 or higher toxicity had a least one or more decompensation event compared with those patients whose patient laboratory values were within normal range. Previous local/regional therapy showed statistically significant differences at the *P* < .05 level when comparing across baseline grades. Most patients whose baseline laboratory values correspond to toxicity grades 0-2 were treated with transcatheter arterial chemoembolization before ICI initiation, while most patients with baseline laboratory values equivalent to grade 3 and 4 toxicities did not receive previous local or regional therapy.

**TABLE 1.** Baseline Patient Characteristics Stratified by Highest Baseline Toxicity Grade (ALT, AST, or TBIL)

Feature	Grade 0 (n = 168)	Grade 1 (n = 291)	Grade 2 (n = 157)	Grade 3 (n = 62)	Grade 4 (n = 4)	*P*
Age, years, median (IQR)	69.8 (65.5-73.3)	67.5 (63.5-71.2)	66.1 (63.3-69.6)	67.3 (63.3-69.8)	65.7 (61.1-69.8)	<.001
Charlson index, median (IQR)	3 (2-4)	3 (2-4)	3 (2-4)	3 (2-4)	3 (2.8-3.3)	.11
Reported sex, No. (%)						
Female	0	3 (1)	1 (0.6)	0	0	.67
Male	168 (100)	288 (99)	156 (99.4)	62 (100)	4 (100)	
Race, No. (%)						
American Indian/Alaska Native	0	0	1 (0.6)	2 (3.2)	0	.14
Asian	0	2 (0.7)	2 (1.3)	2 (3.2)	0	
Black/African American	56 (33.3)	75 (25.8)	39 (24.8)	21 (33.9)	1 (25)	
Native Hawaiian/Pacific Islander	2 (1.2)	4 (1.4)	2 (1.3)	0	0	
White	102 (60.7)	193 (66.3)	105 (66.9)	32 (51.6)	3 (75)	
Unknown	8 (4.8)	17 (5.8)	8 (5.1)	5 (8.1)	0	
Ethnicity, No. (%)						
Hispanic or Latino	12 (7.1)	34 (11.7)	16 (10.2)	8 (12.9)	0	.89
Not Hispanic or Latino	152 (90.5)	252 (86.6)	138 (87.9)	53 (85.5)	4 (100)	
Unknown	4 (2.4)	5 (1.7)	3 (1.9)	1 (1.6)	0	
BMI, No. (%)						
<18.5	5 (3)	13 (4.5)	1 (0.6)	2 (3.2)	0	.41
18.5-24.9	64 (38.1)	101 (34.7)	49 (31.2)	18 (29)	2 (50)	
25-29.9	61 (36.3)	103 (35.4)	55 (35)	26 (41.9)	2 (50)	
30-39.9	36 (21.4)	64 (22)	46 (29.3)	12 (19.4)	0	
≥40	2 (1.2)	10 (3.4)	6 (3.8)	4 (6.5)	0	
Baseline FIB-4,^a^ No. (%)						
<1.45	25 (14.9)	11 (3.8)	5 (3.2)	2 (3.2)	0	<.001
1.45-3.25	91 (54.2)	75 (25.8)	17 (10.8)	4 (6.5)	0	
>3.25	41 (24.4)	183 (62.9)	125 (79.6)	53 (85.5)	3 (75)	
Unknown	11 (6.5)	22 (7.6)	10 (6.4)	3 (4.8)	1 (25)	
Child-Pugh score, No. (%)						
A5	149 (88.7)	235 (80.8)	108 (68.8)	39 (62.9)	1 (25)	<.001
A6	17 (10.1)	43 (14.8)	39 (24.8)	12 (19.4)	0	
B7	2 (1.2)	10 (3.4)	8 (5.1)	7 (11.3)	2 (50)	
B8	0	3 (1)	2 (1.3)	4 (6.5)	1 (25)	
Baseline MELD, No. (%)						
≤9	133 (79.2)	246 (84.5)	128 (81.5)	49 (79)	1 (25)	<.001
10-19	33 (19.6)	43 (14.8)	27 (17.2)	10 (16.1)	1 (25)	
20-29	2 (1.2)	2 (0.7)	2 (1.3)	3 (4.8)	2 (50)	
Baseline MELD-NA, No. (%)						
≤9	133 (79.2)	246 (84.5)	128 (81.5)	49 (79)	1 (25)	<.001
10-19	33 (19.6)	43 (14.8)	27 (17.2)	10 (16.1)	1 (25)	
20-29	2 (1.2)	2 (0.7)	2 (1.3)	3 (4.8)	2 (50)	
History of hepatitis B infection, No. (%)						
No	101 (60.1)	180 (61.9)	87 (55.4)	36 (58.1)	0	.39
Yes	17 (10.1)	24 (8.2)	14 (8.9)	5 (8.1)	1 (25)	
Unknown	50 (29.8)	87 (29.9)	56 (35.7)	21 (33.9)	3 (75)	
History of hepatitis C infection, No. (%)						
No	35 (20.8)	50 (17.2)	28 (17.8)	12 (19.4)	1 (25)	.71
Yes	115 (68.5)	218 (74.9)	121 (77.1)	45 (72.6)	3 (75)	
Unknown	18 (10.7)	23 (7.9)	8 (5.1)	5 (8.1)	0	
History of decompensation events						
Before ICI initiation,^b^ No. (%)						
None	122 (72.6)	163 (56)	65 (41.4)	24 (38.7)	1 (25)	<.001
Varices	22 (13.1)	73 (25.1)	60 (38.2)	18 (29)	1 (25)	
Pulmonary hypertension	20 (11.9)	49 (16.8)	51 (32.5)	17 (27.4)	2 (50)	
Ascites	18 (10.7)	44 (15.1)	52 (33.1)	17 (27.4)	3 (75)	
Portal vein thrombosis	9 (5.4)	31 (10.7)	20 (12.7)	13 (21)	2 (50)	
Hepatic encephalopathy	5 (3)	25 (8.6)	30 (19.1)	9 (14.5)	2 (50)	
Spontaneous bacterial peritonitis	0	8 (2.7)	5 (3.2)	2 (3.2)	1 (25)	
Hepatorenal syndrome	0	3 (1)	0	1 (1.6)	1 (25)	
Baseline AFP values, No. (%)						
<400 ng/mL	75 (44.6)	126 (43.3)	67 (42.7)	28 (45.2)	0	.34
≥400 ng/mL	32 (19)	82 (28.2)	39 (24.8)	15 (24.2)	2 (50)	
Unknown	61 (36.3)	83 (28.5)	51 (32.5)	19 (30.6)	2 (50)	
Previous local/regional therapy,^b^ No. (%)						
TACE	95 (56.5)	168 (57.7)	96 (61.1)	27 (43.5)	1 (25)	.03
Y90	8 (4.8)	21 (7.2)	7 (4.5)	1 (1.6)	0	
RFA-PERC	37 (22)	60 (20.6)	29 (18.5)	9 (14.5)	0	
RFA-LAP	6 (3.6)	15 (5.2)	6 (3.8)	1 (1.6)	0	
CHEMO	52 (31)	106 (36.4)	50 (31.8)	17 (27.4)	1 (25)	
Resection	19 (11.3)	20 (6.9)	11 (7)	0	1 (25)	
XRT	34 (20.2)	45 (15.5)	18 (11.5)	5 (8.1)	0	
IMRT	12 (7.1)	17 (5.8)	4 (2.5)	4 (6.5)	0	
SBRT	11 (6.5)	7 (2.4)	1 (0.6)	1 (1.6)	0	
TRANSPLANT	0	0	1 (0.6)	0	0	
None	52 (31)	86 (29.6)	50 (31.8)	33 (53.2)	3 (75)	
Previous targeted therapy,^c^ No. (%)						
No	42 (25)	54 (18.6)	33 (21)	20 (32.3)	1 (25)	.33
Yes	121 (72)	232 (79.7)	121 (77.1)	42 (67.7)	3 (75)	
Unknown	5 (3)	5 (1.7)	3 (1.9)	0	0	

NOTE. Age, FIB-4, eCTP, MELD/MELD-NA, previous decompensation events, and previous local regional therapy demonstrate statistically significant differences between baseline toxicity grades.

Abbreviations: AFP, alpha-fetoprotein; CHEMO, chemotherapy; eCTP, estimated Child-Turcott-Pugh; FIB-4, Fibrosis-4 score; HCC, hepatocellular carcinoma; ICD, International Classification of Diseases; ICI, immune checkpoint inhibitor; IMRT, intensity-modulated external-beam radiation therapy; MELD, model for end-stage liver disease; MELD-NA, Model for End Stage Liver Disease-Sodium Score; PLT, platelet count; RFA-LAP, laparoscopic radiofrequency ablation; RFA-PERC, percutaneous radiofrequency ablation; SBRT, stereotactic body radiation therapy; TACE, transcatheter arterial chemoembolization; TBIL, bilirubin; TRANSPLANT, liver transplant; VAR, varices; XRT, external-beam radiation therapy; Y90, yttrium-90 radioembolization.

a Baseline FIB-4 score unavailability reflects the requirement that PLT and ALT/AST be derived from laboratory reports within 14 days of one another and most proximate to and within 42 days before the initiation of ICI.

b May sum to larger than the stratum of the cohort as the same patient may be in multiple strata, for example, a patient may have multiple types of decompensation events or previous therapy.

c Unknown previous targeted therapy exposure reflects the requirement that drug era be within ±7 days of an encounter coded with an ICD-9/10 code for HCC.

### Interannotator Agreement

Sixty-three of 682 patients were selected for annotation to create a balanced sample of patients based on the number of patients determined to be irAEs, no irAEs, and indeterminant status by an initial version of the algorithm (see Table 2, see the Data Supplement, Methods: Chart review, for additional details). Of these patients, expert adjudicators agreed on the irAE status of 44 of 63 patients (69.8%, Fleiss' kappa: 0.434). Annotators agreed on the date of first dose of ICI, defined as a date difference between reviewers of 7 days or less, in 55 of 63 patients (87.3% concordance with a median absolute difference of 0 days and IQR of [0-2] days).

**TABLE 2.** Interannotator Agreement Including Hepatic + Nonhepatic irAEs

Feature Adjudicated	No.	Concordance	*P*
irAE, No. (%) (Fleiss' kappa)	63	44 (69.8) (0.434)	<.001
First ICI, No. (%) (med. abs difference, days [IQR])	63	55 (87.3) (0 [0-2])	—
Adverse event date, No. (%) (med. abs difference, days [IQR])	19	8 (42.1) (8.5 [0-40.75])	—
Type of adverse event, No. (%)	19	18 (94.7)	—
Grade, No. (%)	19	9 (47.4)	—
Laboratory, No. (%)	15	14 (93.3)	—
Response to event, No. (%)	19	18 (94.7)	—
Date of steroid initiation, No. (%) (med. abs difference, days [IQR])	17	9 (52.9) (4 [1.25-19.25])	—

NOTE. Two clinician-adjudicators reviewed 63 patients for chart review. Reviewers agreed on irAE status 69.8% of the time, leading to a Fleiss' kappa of 0.434. Interannotator agreement exceeded 85% for date of first ICI (where agreement was defined as a date within 7 days), adverse event type, and the laboratory value presenting the irAE. However, reviewers only achieved agreement of 42.1%, 47.4%, and 52.9% for the adverse event date, grade of severity, and date of treatment, respectively. Disagreements include cases in which one reviewer did not provide a response; quantiles reflect provided numerical inputs.

Abbreviations: ICI, immune checkpoint inhibitor; irAE, immune-related adverse event.

### Algorithm Performance

Performance results and confusion matrices are presented in Table 3 (see extended results in the Data Supplement, Table S3, for additional details). Of 63 adjudicated patients, 25 were determined to be irAEs of any CTCAE grade or type, of which 16 were determined to be hepatic irAEs, two were determined to be indeterminant, and the remaining 36 patients did not present any irAE based on chart review consensus.

**TABLE 3.** Algorithm Performance and Confusion Matrices

irAE Algorithm															
Hepatic irAEs															
Feature	Value	No.	Con.	TP	FP	TN	FN	Prev.	Sp.	Sens.	PPV	Eff.	F1	W.F1	Macro.F1
irAE	All	63	47 (74.6)	—	—	—	—	—	—	—	—	—	—	0.74	0.47
	Yes	16	10 (62.5)	10	9	38	6	0.25	0.81	0.63	0.53	0.76	0.57	—	—
	No	45	37 (84.4)	37	7	11	8	0.71	0.61	0.84	0.84	0.78	0.84	—	—
	Ind.	2	0	0	0	61	2	0.03	1	0	—	0.97	—	—	—

All-Comers															
Hepatic irAEs															
Feature	Value	No.	Con.	TP	FP	TN	FN	Prev.	Sp.	Sens.	PPV	Eff.	F1	W.F1	Macro.F1
irAE	All	63	26 (41.3)	—	—	—	—	—	—	—	—	—	—	0.39	0.28
	Yes	16	16 (100)	16	37	10	0	0.25	0.21	1	0.30	0.41	0.46	—	—
	No	45	10 (22.2)	10	0	18	35	0.71	1	0.22	1	0.44	0.36	—	—
	Ind.	2	0	0	0	61	2	0.03	1	0	—	0.97	—	—	—

NOTE. Here, the irAE algorithm is compared with the all-comers approach, in which any elevation of ALT, AST, or TBIL is inferred to be an irAE (an algorithm selected for sensitivity = 1).

Abbreviations: Con., concordance; Eff., efficiency (accuracy); FN, false negative; FP, false positive; irAE, immune-related adverse event; Macro.F1, macro-weighted F1 score; PPV, positive predictive value; Prev., prevalence; Sens., sensitivity; Sp., specificity; TN, true negative; TP, true positive; W.F1, prevalence-weighted F1 score.

The algorithm achieves a weighted F1 score of 0.74, weighted by prevalence of irAEs. For detection of hepatic irAEs, the specificity of the algorithm is 0.81 and sensitivity is 0.63 with a resulting positive predictive value (PPV) of 0.53, negative predictive value (NPV) of 0.82, and a test efficiency of 0.76.^24^ In the context of hepatic irAEs, the algorithm achieves a percent concordance of 74.6%, which corresponds to Fleiss' kappa of agreement with the ground truth (consensus of expert opinion) of 0.40 (*P* < .001). The algorithm correctly identifies seven of nine available event dates for hepatic irAEs (77.8%); 10 of 10 types of laboratory values associated with the event (100%); and seven of 10 (70%) grades of severity, including one of two (50%) CTCAE grade 4 irAEs; five of six (83.3%) of grade 3 irAEs; and one of two (50%) of grade 2 irAEs. By comparison, the algorithm achieves a specificity of 0.82 and sensitivity of 0.44 for detection of any irAE with a resulting PPV of 0.63 and NPV of 0.68 with an overall efficiency of 0.68 and prevalence-weighted F1 score of 0.65.

By contrast, the all-comers algorithm achieves sensitivities of 1 and 0.92 for hepatic and nonhepatic irAEs, respectively, with a specificity of 0.21 for both hepatic and any irAE. The approach identifies the correct event date in 10 of 15 (66.7%) of hepatic irAEs, the correct type of laboratory value in 14 of 16 detected patients (87.5%), and the correct grade in 12 of 16 detected patients (75%), including one of one grade 1 hepatic irAEs (100%), one of three grade 2 detected hepatic irAEs (33.3%), nine of 10 (90%) grade 3 detected hepatic irAEs, and one of two (50%) grade 4 hepatic irAEs correctly detected (see the Data Supplement, Results, Table S3 and Fig S4, for additional details).

### Error Analysis

We undertook a root cause analysis to understand the performance of the algorithm. Of six irAEs misclassified as non-irAEs, three of six (50%) were events corresponding to CTCAE grade 1 severity, below the intended design specifications of the algorithm. Of these, one was misclassified because of the date of the adverse event relative to ICI initiation and two because of the lack of steroid treatment, consistent with the clinical management of grade 1 hepatic irAEs.^1,6,8,11^ One grade 2 irAE was misclassified because of an initial date greater than allowed by our rules and one grade 2 event was misclassified because of no use of steroids, which may reflect irAE management and differences in clinicians' choice of management with additional knowledge of the patient's history.^1,6,8,11^ One grade 3 irAE was misclassified because of a steroid treatment duration that exceeded the duration selected in the rules, which reflects the wide and varied presentation of immune-mediated hepatitis (see the Data Supplement, Results: Patient-informed kinetics, Extended patient characteristics, and Analysis of false positives, for additional details).

### Downstream Applications of irAE Detection/irAE Incidence

We first used our algorithm to calculate the incidence of immune-mediated hepatitis by type (see the Data Supplement, Results: Incidence of irAEs in the cohort, and the Data Supplement, Table S4, for additional details).

Patients who experienced any grade of irAE had significantly higher peak NLRs (median, 9.2 [IQR, 7.2-18.3]) compared with those patients who did not experience an irAE (median, 5.6 [IQR, 3.4-10.5]) during 0-14 weeks of treatment (Table 4).

**TABLE 4.** Patient Characteristics Associated With Detected irAEs (all patients determined by the algorithm to be irAEs)

Feature	Experienced irAE (n = 30)	No irAE (n = 651)	Indeterminant (n = 1)	*P*
Max grade at baseline, No. (% stratum)				
Grade 0	6 (20)	162 (24.8)	0	.66
Grade 1	12 (40)	279 (42.8)	0	
Grade 2	7 (23.3)	150 (23)	1 (100)	
Grade 3	5 (16.7)	57 (8.7)	0	
Grade 4	0	4 (0.6)	0	
Previous targeted therapy, No. (% stratum)				
No	6 (20)	143 (22)	1 (100)	.37
Yes	24 (80)	495 (76)	0	
Unknown	0	13 (2)	0	
Peak eosinophil to lymphocyte ratio 0-14 weeks on ICI				
No. (% stratum)	22 (73.3)	502 (77.1)	1 (100)	—
Median (IQR)	0.3 (0.2-0.5)	0.2 (0.1-0.4)	0.1 (0.1-0.1)	.28
Peak neutrophil to lymphocyte ratio 0-14 weeks on ICI				
No. (% stratum)	21 (70)	533 (81.9)	1 (100)	—
Median (IQR)	9.2 (7.2-18.3)	5.6 (3.4-10.5)	7 (7-7)	.002
ELR variability 0-14 weeks on ICI, No. (% stratum)				
Quartile 1	6 (20)	124 (19.0)	1 (100)	.14
Quartile 2	4 (13.3)	127 (19.5)	0	
Quartile 3	2 (6.7)	129 (19.8)	0	
Quartile 4	10 (33.3)	122 (18.7)	0	
NLR variability 0-14 weeks on ICI, No. (% stratum)				
Quartile 1	1 (3.3)	138 (21.2)	0	.08
Quartile 2	5 (16.7)	133 (20.4)	0	
Quartile 3	5 (16.7)	134 (20.6)	0	
Quartile 4	10 (33.3)	128 (19.7)	1 (100)	

NOTE. Peak NLR in the first 14 weeks of ICI treatment was significantly higher in those patients who were determined to have experienced a hepatic irAE than those who did not at the level of *P* < .05. NLR variability was also observed to be higher in those patients who experienced irAEs but did not rise to the level of significance of *P* < .05. ELR and NLR counts reflect availability of data adhering to (1) baseline measurements and (2) temporal definitions in the first 14 weeks of treatment.

Abbreviations: ELR, eosinophil to leukocyte ratio; ICI, immune checkpoint inhibitor; irAEs, immune-related adverse events; NLR, neutrophil to lymphocyte ratio.

## DISCUSSION

Overall, the performance of the algorithm is comparable with the agreement of individual clinician experts when evaluating retrospective patients for hepatic irAEs. Clinical domain experts achieve somewhat higher concordance when identifying irAEs compared with the algorithm, 69.8% compared with 62.5%, but overall agreement with the ground truth, as indicated by the test efficiency is 76.2% exceeding the concordance of annotators. Moreover, the algorithm exceeds concordance with annotators for hepatic irAEs on the bases of the presenting laboratory value, events of grade 2 or 3 severity, and the grade of severity. The algorithm exceeds interannotator concordance in considerations of the date of the adverse event. Although some of these performance comparisons may represent typographical errors on the part of annotators, such factors would also be present in any case of chart abstraction and highlight some of the advances to implementing automated irAE detection.

The algorithm achieves high specificity (0.81) and NPV (0.84) but modest sensitivity (0.63) and PPV (0.53) for detection of hepatic irAEs. Although not intended as a causality assessment tool for clinical decision making, to provide context for these performance metrics, recent drug-induced liver toxicity algorithms based on the RUCAM assessment report PPVs between 0.01 and 0.402.^25,26^ Thus, in comparison, our phenotype achieves a PPV 31.8% greater than EHR-based implementations of the RUCAM. Although distinct in their development and purpose, the most applicable comparison between the performance of our tool and the performance of the RECAM is the comparison of concordance with expert reviewers (see the Data Supplement, Results: Comparison of irAE algorithm to the concordance of clinical experts, and the Data Supplement, Fig S4, for additional details). In our case, we compare the consensus of expert opinion with the phenotype result. The RECAM achieves a percent concordance with reviewers in the final round of development of 62.4%,^15^ while the phenotype presented here for irAEs achieves 74.6% concordance with the consensus of expert reviewers.

In comparison, we also evaluated an all-comers algorithm to mimic the approach used in some randomized controlled trials of ICI^1^ and to mimic a design tuned to sensitivity of 1.^25,26^ We see while this approach does indeed detect all true patients with irAEs, it does so with the expected loss of specificity. As severe irAEs are relatively rare, this loss of specificity may be particularly detrimental for applications of case screening where the algorithm may overestimate the caseload requiring subsequent screening. This contrasts with the use case of a clinical trial in which the clinician may have additional knowledge of patient history to attribute elevations in ALT, AST, and TBIL to ICI.

Patient characteristics highlight the difficulty in identifying irAEs by routine laboratory biomarkers as well as justify the focus on the temporal relationship between elevations in AST, ALT, and TBIL, and prescribing patterns of steroids associated with the clinical management of irAEs.

Despite the modest PPV, our approach identifies NLR as a distinguishing marker of irAE, supported by previous studies^9^ and suggests that there is no direct relationship between exposure to TKI therapy before ICI and the development of irAEs.

Previous studies used emergent encounters and steroid treatment to identify ICI-mediated irAEs to examine outcomes.^27,28^ NLR and detection of irAEs was found to be prognostic of irAEs, not necessarily hepatic in nature, but in a cohort of patients in which irAEs were identified by clinician adjudication.^12^ Here, we demonstrate a computable hepatic irAE phenotype using only routine laboratory components and medication orders extracted from the EHR. The output of our phenotype (1) can be useful in large-scale EHR-based studies to identify hepatic irAEs where chart abstraction would be unfeasible, (2) includes considerations of onset timing relevant to the extended courses associated with ICI therapy (>90 days after initiation), and (3) if desired, summarizes and can provide output of time parameters to streamline chart review.

As with any cohort study, our phenotype has several limitations. It was developed using a cohort of patients from the VHA, which is composed predominantly of male patients, which may limit its applicability to other cohorts or study populations. Our cohort inclusion criteria included a diagnosis of HCC and, as such, may not be applicable to cohorts of patients with different diagnoses. Future work will examine its applicability to other diagnoses. For our purposes, we only seek to identify hepatic irAEs. However, patients may still experience a range of nonhepatic irAEs. We recognize that our phenotype has modest performance as a tool intended to facilitate retrospective identification of irAEs and not intended as a tool for clinical decision making.

In conclusion, we developed a rule-based algorithm to retrospectively identify hepatic irAEs in a national cohort of patients with HCC using continuous monitoring of routine biomarkers. Our approach achieves comparable performance to clinical experts and provides a framework for facilitating clinician review. We obtain modest PPV that improves upon reported RUCAM results by 31.8% and has a specificity of 0.81 and overall efficiency of 0.76 despite using only biomarkers that are neither sensitive nor specific to irAEs.
